# Humoral response to mRNA-based COVID-19 vaccine and booster effect of a third dose in patients with mature T cell and NK-cell neoplasms

**DOI:** 10.1007/s00277-023-05142-4

**Published:** 2023-03-02

**Authors:** Mirei Kobayashi, Akio Mori, Masahiro Onozawa, Shihori Tsukamoto, Hajime Senjo, Takashi Ishio, Emi Yokoyama, Minoru Kanaya, Koh Izumiyama, Makoto Saito, Haruna Muraki, Masanobu Morioka, Takanori Teshima, Takeshi Kondo

**Affiliations:** 1 Blood Disorders Center, Aiiku Hospital, Sapporo, Japan; 2 Department of Hematology, Faculty of Medicine, Hokkaido University, Sapporo, Japan; 3 Division of Laboratory, Aiiku Hospital, Sapporo, Japan; 4 Sapporo Clinical Laboratory Inc, Sapporo, Japan

**Keywords:** COVID-19, SARS-CoV-2, Mature T cell and NK-cell neoplasms, Vaccine, Booster vaccine effect

## Abstract

Patients with lymphoid malignancies have impaired humoral immunity caused by the disease itself and its treatment, placing them at risk for severe coronavirus disease-19 (COVID-19) and reduced response to vaccination. However, data for COVID-19 vaccine responses in patients with mature T cell and NK-cell neoplasms are very limited. In this study of 19 patients with mature T/NK-cell neoplasms, anti-severe acute respiratory syndrome coronavirus-2 (SARS-CoV-2) spike antibodies were measured at 3 months, 6 months, and 9 months after the second mRNA-based vaccination. At the time of the second and third vaccinations, 31.6% and 15.4% of the patients were receiving active treatment. All patients received the primary vaccine dose and the third vaccination rate was 68.4%. In patients with mature T/NK-cell neoplasms, both seroconversion rate (*p* < 0.01) and antibody titers (*p* < 0.01) after the second vaccination were significantly lower than those in healthy controls (HC). In individuals who received the booster dose, patients had significantly lower antibody titers than those in HC (*p* < 0.01); however, the seroconversion rate in patients was 100%, which was the same as that in HC. The booster vaccine resulted in a significant increase of antibodies in elderly patients who had shown a response that was inferior to that in younger patients after two doses of vaccination. Since higher antibody titers and higher seroconversion rate reduced the incidence of infection and mortality, vaccination more than three times may have the advantage for patients with mature T/NK-cell neoplasms, especially in elderly patients. Clinical trial registration number: UMIN 000,045,267 (August 26th, 2021), 000,048,764 (August 26th, 2022).

**Supplementary Information:**

The online version contains supplementary material available at 10.1007/s00277-023-05142-4.

## Introduction

Severe acute respiratory syndrome coronavirus 2 (SARS-CoV-2), the virus responsible for coronavirus disease-19 (COVID-19), has had devastating consequences globally and the end of the outbreak is not yet in sight. mRNA-based vaccines, BNT162b2 or mRNA-1273, were developed and these are useful in preventing infection and disease severity from COVID-19 [1–3]. However, previous pivotal studies conducted on only healthy individuals and did not include patients with hematological malignancies [1, 2, 4–6]. Therefore, data for COVID-19 vaccine responses in patients with hematological malignancies, particularly mature T cell and NK-cell neoplasms, are limited. In contrast, previous studies have reported that patients with hematological malignancies have a high mortality rate from COVID-19 [7–14]. Patients with hematological malignancies have impaired humoral immunity secondary to their malignancy and its treatment, placing them at risk for severe COVID-19 and reduced response to vaccination [15–18]. Furthermore, recent studies have reported that patients with hematological malignancies have lower seroconversion rate compared to that in healthy controls (HC) [19–25]. Homologous and heterologous booster vaccines with BNT162b2, mRNA-1273, and Ad26.COV2.S were shown to be immunogenic in healthy adults [26]. In contrast, the booster vaccine efficacy in patients with hematological malignancies seems to be inferior to that in healthy individuals [27–31]. In this study, we measured the antibody titers of COVID-19 in patients with mature T/NK-cell neoplasms who received more than at least two doses of an mRNA-based COVID-19 vaccine and compared them to those in HC. We also investigated the antibody titers against SARS-CoV2 in patients with mature T/NK-cell neoplasms who received the third mRNA-based COVID-19 vaccine dose (booster).

## Patients and methods

### Patients

Patients with mature T/NK-cell neoplasms under follow up at the Blood Disorders Center at Aiiku Hospital during the period from May 26, 2021, to August 5, 2022, were included in this study. All of the patients were vaccinated with at least two doses of an mRNA-based COVID-19 vaccine, either BNT162b2 or mRNA-1273. BNT162b2 and mRNA-1273 were administered 21 and 28 days apart, respectively. All disease statuses of patients were determined at the time of the second vaccination. We recruited healthcare workers aged 50 years or older who had received at least two doses of BNT162b2 vaccine as HC. They were unlikely to be transmitted from inpatients since our hospital had not accepted COVID-19 patients. Individuals with a known history of COVID-19 were excluded from both cohorts of patients and HC. This study was a part of a prospective observation study (UMIN 000,045,267, 000,048,764) and it was conducted in compliance with ethical principles based on the Helsinki Declaration and was approved by the institutional review board of Aiiku Hospital. All patients provided written informed consent.

### Assessment of serological response

Anti-SARS-CoV-2 spike (S) immunoassays were performed at 3 months ± 4 weeks, 6 months ± 4 weeks, and 9 months ± 4 weeks after the second vaccine dose as previously described [32–35]. This assay has a minimum measurement value of 0.4 U/ml and a maximum measurement value of 25,000 U/ml. A value of 0.8 U/ml or higher is considered as a positive result [36]. An antibody titer less than 0.4 U/ml or more than 25,000 U/ml was calculated as 0.4 U/ml or 25,000 U/ml for convenience, respectively. All individuals who received a third dose were administered the third dose between 3-month and 9-month blood samplings, and the choice of cross-vaccination was made available.

### Statistical analysis

Median antibody titers in paired samples and in unpaired samples were compared using the Wilcoxon signed-rank test and the Mann–Whitney *U* test, respectively. Differences between the means of two different groups were analyzed using the *t*-test. We used Fisher’s exact test for categorical variables. *p* values less than 0.05 for statistical analyses were considered significant. All statistical analyses were performed with EZR (Jichi Medical University, Saitama, Japan) [37].

## Results

### Characteristics of patients and healthy controls

Nineteen patients with mature T/NK-cell neoplasms whose median age was 74 (range: 43–87) years were enrolled in this study. The characteristics of the patients are shown in Table 1. The patients included 6 patients with angioimmunoblastic T cell lymphoma (AITL), 5 patients with peripheral T cell lymphoma, not otherwise specified (PTCL-NOS), 2 patients with anaplastic large cell lymphoma (ALCL), 2 patients with nodal peripheral T cell lymphoma with T follicular helper phenotype (PTCL with Tfh phenotype), one patient with adult T cell leukemia/lymphoma (ATL/L), one patient with T cell large granular lymphocytic leukemia (T-LGLL), one patient with extranodal NK/T cell lymphoma, nasal type (ENKL), and one patient with monomorphic epitheliotropic intestinal T cell lymphoma (MEITL). HC included 29 individuals with a median age of 55 (50–72) years, and 62.1% were female. None of the patients with mature T/NK-cell neoplasms following at our hospital had a history of SARS-CoV-2 infection during the follow-up period.

**Table 1:** Patients’ characteristics

	Mature T-cell and NK-cell neoplasms (*n* = 19)	
Age median (range)	74 (43–87)	
Sex male/female	9/10	
Period from diagnosis to second vaccination, months, median (range)	23 (0–176)	
Period from second dose to third dose*, days, median (range)	228 (182–315)	
	First/second dose	Third dose
Patients with seroconversion, *n* (%)	14/19 (73.7)	13/13 (100)
On active therapy at the time of vaccination		
Yes	6	2
CHOP	1	0
CHOEP	1	0
Romidepsin monotherapy	1	0
Etoposide monotherapy	0	1
Prednisolone + cyclosporine	1	0
Prednisolone monotherapy	2	1
No	13	11
Treatment off in CR	10	10
Terminated active treatment	1	1
Previously untreated (treatment-naïve)	2	0
Status at the time of vaccination		
CR	12	10
PR	3	2
SD	1	1
Relapse/refractory	1	0
Previously untreated (treatment-naive)	2	0
Vaccination rate, n (%)	19/19 (100)	13/19 (68.4)
Vaccine subtype		
BNT162b2	17	8
mRNA-1273	2	5
Not yet vaccinated	0	6

*Patients who did not receive the third dose were excluded. Abbreviations: CHOP, (cyclophosphamide, doxorubicin, vincristine, and prednisolone); CHOEP, CHOP plus etoposide; CR, complete response; PR, partial response; SD, stable disease

### Seroconversion rates and antibody titers at 3 months after the second vaccination

**Fig. 1:**
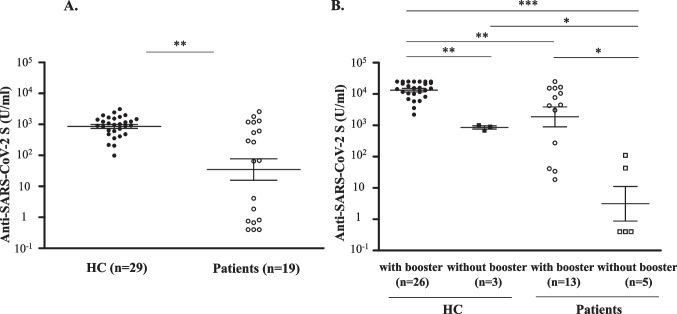
(**A**) Anti-SARS-CoV-2 S antibody titers at 3 months after the second vaccination in healthy controls and in patients with mature T/NK-cell neoplasms. (**B**) Anti-SARS-CoV-2 S antibody titers at 9 months after the second vaccination in healthy controls and patients with mature T/NK-cell neoplasms who received the booster vaccine or those who did not receive the booster vaccine. The Mann–Whitney *U* test was used to compare medians of antibody titers. The two short lines show interquartile range (IQR) and the center long line shows the median. HC, healthy controls

Six of the 19 patients were receiving treatment at the time of the second vaccination. The seroconversion rate after the second vaccination in patients was 73.7%, which was significantly lower than that in HC (100%) (*p* < 0.01). At 3 months after the second vaccination, patients showed a significantly lower antibody titer than that in HC (67.8 (interquartile range (IQR): 0.8–893.0) U/ml vs. 974.0 (608.0–1466.0) U/ml, *p* < 0.01) (Fig. 1A).

### Seroconversion rates and antibody titers after the booster dose

Third vaccination rates were 89.7% and 68.4% in HC and patients, respectively. Two of the 13 patients who received the third dose were receiving active treatment at the time of the third vaccination. Seroconversion rates after the third vaccination in patients and HC were both 100%.

Next, we examined seroconversion rates at 9 months in patients who received the booster vaccine (*n* = 13) and those who did not receive the booster vaccine (*n* = 5). The seroconversion rate at 9 months was significantly higher in patients who received the booster vaccine than in patients who did not receive the booster vaccine (100% vs. 40.0%, *p* < 0.05). The antibody titer in patients who did not receive the booster dose (0.4 (0.4–42.7) U/ml vs. 15,324.0 (10,122.5–24,361.3) U/ml, *p* < 0.001) and that in patients who received the booster dose (4515.0 (273.0–15,373.0) U/ml, *p* < 0.01) at 9 months were both lower than that in healthy individuals who received the booster dose (Fig. 1B). Furthermore, patients who received the booster vaccine had a significantly higher median antibody titer at 9 months than that in patients who did not receive the booster vaccine (4515.0 (273.0–15,373.0) U/ml vs. 0.4 (0.4–42.7) U/ml, *p* < 0.05) (Fig. 1B). The mean age of the five patients who did not receive the booster vaccine was 62.2 (± 15.8) years old. In contrast, the mean age of the 13 patients who had received the booster vaccine was 74.2 (± 9.9) years old, which was older than that of the patients who had not received the booster vaccine although the difference was not significant (*p* = 0.0692).

 In contrast, four of the five patients who had not received the booster vaccine were receiving active treatment at the time of the second vaccination. The other patient who was not receiving active treatment at the time of the second vaccination had a low antibody titer of less than 0.4 U/ml at 9 months. Only one of the 13 patients who received the booster vaccine was receiving active treatment at the time of the second vaccination. The antibody titer of this patient at 9 months was relatively high at 4500 U/ml.

### Time-dependent decrease in antibody titer and booster vaccine response

**Fig. 2:**
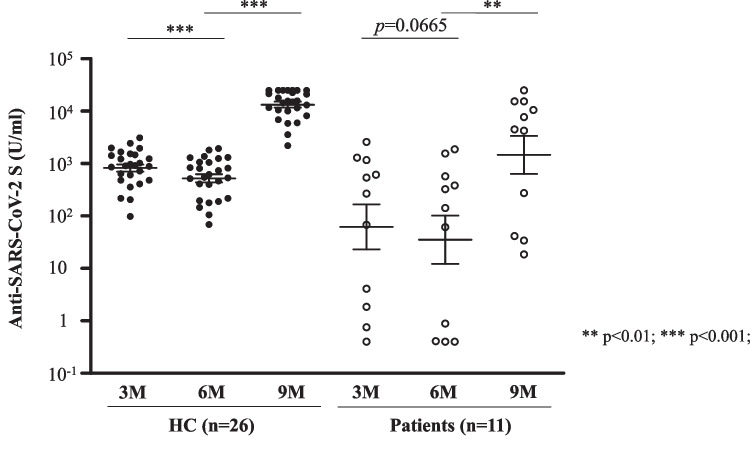
Anti-SARS-CoV-2 S antibody titers after the initial vaccination and booster vaccination in healthy controls and patients with mature T/NK-cell neoplasms. Individuals who did not receive a third dose were excluded from this analysis. The Wilcoxon signed-rank test was used to compare medians of antibody titers in paired individuals. The two short lines show interquartile range (IQR) and the center long line shows the median. HC, healthy controls. M, months

The median antibody titers in 11 patients and 26 healthy individuals who had all 3-month, 6-month, and 9-month paired samples and who also received the third dose were compared using the Wilcoxon signed-rank test. Since patients who received third dose before the 6-month blood sampling were excluded, all third doses were administered between 6-month and 9-month blood samplings in this analysis. In HC, the median antibody titer at 6 months was significantly lower than that at 3 months (582.5 (263.3–1067.0) U/ml vs. 913.5 (514.3–1456.3) U/ml, *p* < 0.001). Even in patients, the median antibody titer at 6 months was also lower than that at 3 months, but the difference was marginally tendency (*p* = 0.0665). After the booster vaccine, there was a significant increase in antibody titers in both HC (582.5 (263.3–1067.0) U/ml vs. 15,324.0 (10,122.5–24,361.3) U/ml, *p* < 0.001) and patients (142.0 (0.7–478.0) U/ml vs. 4515.0 (157.3–12,938.5) U/ml, *p* < 0.01) (Fig. 2).

### Factors affecting vaccine responses in patients with mature T/NK-cell neoplasms

**Fig. 3:**
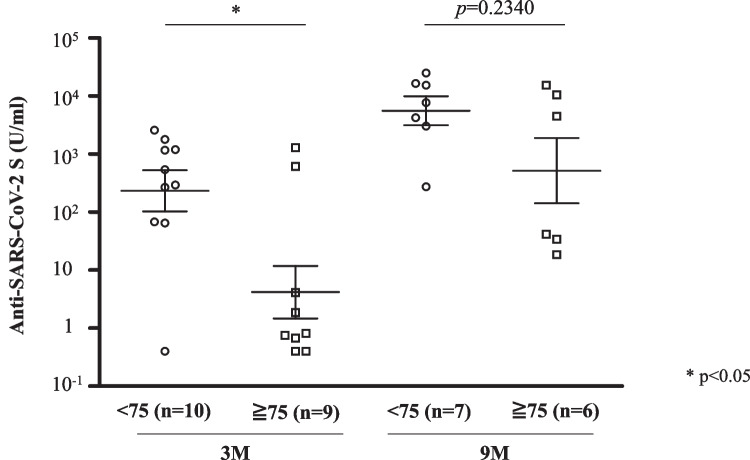
Anti-SARS-CoV-2 S antibody titers at 3 months and 9 months after the second vaccination in patients 75 years of age or older and in patients younger than 75 years of age. Individuals who did not receive a third dose prior to the 9-month blood sampling were excluded from the analysis of 9-month blood sampling. The Mann–Whitney *U* test was used to compare medians of antibody titers. The two short lines show interquartile range (IQR) and the center long line shows the median. M, months

Since the median age of the patients in our cohort was 74 years, we divided the patients by age of 75 years for clinical usefulness. The seroconversion rate at 3 months after the second vaccination in patients aged 75 years or older was lower than that in patients younger than 75 years of age, but the difference was not statistically significant (55.6% vs. 90.0%, *p* = 0.1410). After the booster vaccination, the seroconversion rates increased to 100% in both patients younger than 75 years of age and patients aged 75 years or older. In contrast, the median antibody titer at 3 months after the second dose in patients aged 75 years or older was significantly lower than that in patients younger than 75 years of age (0.8 (0.7–4.1) U/ml vs. 415.5 (118.1–1192.0) U/ml, *p* < 0.05) (Fig. 3). Of note, after the booster dose, the median antibody titer in patients aged 75 years or older was comparable to that in patients younger than 75 years of age (2278.3 (36.0–9006.8) U/ml vs. 7679.0 (3632.5–15,961.5) U/ml, *p* = 0.2340).

The vaccine response was examined in 6 patients who were receiving active treatment and 13 patients who were not receiving active treatment at the time of the second vaccination. There was no significant difference in the seroconversion rate at 3 months after the second vaccination between patients who were receiving and those who were not receiving active treatment (66.7% vs. 76.9%, *p* = 1.0000). The median antibody titer in patients receiving active treatment was lower than that in untreated patients at 3 months after the second vaccination, but the difference was not statistically significant (Supplemental Fig. 1). Seroconversion rates after the third vaccination in patients receiving and those not receiving active treatment were both 100%, and there was no significant difference in the median antibody titers after the booster dose between these patients (Supplemental Fig. 1). There were no significant differences in seroconversion rate (complete response (CR) vs. non-CR: 66.7% vs. 100%, *p* = 0.2610 at 3 months, both 100% at 9 months) and antibody titers depending on disease status at the second vaccination both at 3 months and 9 months after the second vaccination (Supplemental Fig. 2).

### Antibody titers in each patient

The dynamics of antibody titers in each patient are shown in Supplemental Fig. 3. Five patients had antibody titers of more than 1000 U/ml at 3 months. Their ages varied from 53 to 85 years old. Four of the five patients remained in CR for more than one year after the end of treatment until the time of initial vaccination. Two of them remained in CR for more than 8 years after the end of treatment until the time of initial vaccination. These two patients had high antibody titers of more than 5000 U/ml at 6 months, but they had received the booster vaccine 5 and 9 days before the day of 6-month blood sampling, respectively. As we mentioned before, these patients were excluded from the Wilcoxon signed-rank analysis in Fig. 2.

## Discussion

In our institution, we routinely recommend COVID-19 vaccination to our patients, especially these with malignant lymphoma who were receiving active treatment. Recent studies have shown that the seroconversion rates after two doses of mRNA-based COVID-19 vaccines were 34–58% in patients with aggressive non-Hodgkin lymphoma (NHL) and 43–61% in patients with indolent NHL [28, 38, 39]. In patients with lymphoid malignancies, the seroconversion rate and antibody titer were significantly lower than those in HC after the second vaccination [39]. In a study of only 8 patients with T cell lymphoma, the seroconversion rate was 75% after two doses of mRNA-based vaccines, which is consistent with our results [39]. Furthermore, the seroconversion rate after a booster vaccine in patients with B-NHL in whom two previous doses were ineffective was 29.5% [30]. Those studies also showed that recent administration of anti-CD20 monoclonal antibodies reduces the vaccine response [28, 30, 39]. Furthermore, in patients with hematological malignancies, the vaccine responses to the primary and booster doses were reported to be lower in elderly patients [20–23, 25, 27–29]. However, data for COVID-19 vaccine responses in patients with mature T/NK-cell neoplasms, especially data for the booster effect, are very limited.

 This study was conducted in patients with mature T/NK-cell neoplasms, and although none of the patients had received anti-CD20 monoclonal antibodies at the time of vaccinations, the seroconversion rate and median antibody titer after two doses were lower than those in HC, as previously reported for patients with B cell malignancies [24, 25, 28, 30, 38, 39]. Furthermore, patients who received the booster dose had lower antibody titers at 9 months than those in healthy individuals who received the booster dose. These results may have been influenced by CHOP (cyclophosphamide, doxorubicin, vincristine, and prednisolone) therapy and other chemotherapy as well as the inherent immunodeficiency of patients with malignant lymphoma itself. However, since the number of patients was small in our study, it seemed to be difficult to perform further sub-analysis for association between treatment and booster vaccine efficacy. On the other hand, since patients who were receiving active treatment tend to have lower antibody titers than those who were not receiving active treatment, we recommend tixagevimab and cilgavimab for these patients [40].

In patients with mature T/NK-cell neoplasms, the seroconversion rate after the second dose was 73.7%, which was significantly lower than that in HC, but the seroconversion rate after the booster dose in the patients was 100%, which was the same as that in HC. In patients with hematological malignancies, although no significant association was found between the seroconversion rate after vaccination and the incidence of COVID-19, no patient with seroconversion died from COVID-19 [28]. Furthermore, as shown in Fig. 2 and Supplemental Fig. 3, it should be noted that the acquired antibody titer decreases in a time-dependent manner. Therefore, vaccination more than three times should be considered in patients with mature T/NK-cell neoplasms.

The median antibody titer in patients aged 75 years or older was significantly lower than that in patients younger than 75 years of age at 3 months after the primary vaccination. However, the median antibody titer after administration of the booster vaccine in patients aged 75 years or older increased to a level comparable to that in patients younger than 75 years of age. Although the preventive antibody titer against SARS-CoV-2 infection is unknown, it has been reported that higher antibody titers contributed to protection against SARS-CoV-2 infection [41, 42]. Therefore, since a booster dose may be more beneficial in elderly patients, administration of a booster vaccine should be considered especially in the elderly.

Our study has several limitations. HC were health care workers. Although our hospital had not accepted COVID-19 patients, the possibility of individuals with asymptomatic COVID-19 cannot be ruled out. The median age of the patient was 74 (range: 43–87) years, whereas the median age of HC was 55 (50–72) years. Thus, there appeared to be differences in the age distribution of patients and HC. This study included a heterogenous patient population and only humoral immunity was evaluated. T cell immunity, which is also important for preventing a severe course of COVID-19, was not evaluated [43]. Our study included both patients who had completed the treatment and those undergoing the treatment. The proportion of patients who were receiving active treatment at the time of the second vaccination in the group of patients who had not received the booster vaccine was higher than the group of patients who had received the booster vaccine. Therefore, as shown in Supplemental Fig. 1, it cannot be ruled out that treatment may have caused an underestimation of antibody titers at 9 months in the group of patients who had not received the booster vaccine. Finally, the number of patients was small. Therefore, our results should be confirmed by further prospective large-scale studies.

In conclusion, in patients with mature T/NK-cell neoplasms, both the seroconversion rate and antibody titer after the second vaccination were significantly lower than those in HC. In contrast, in patients who received the booster dose, the median antibody titer was significantly lower than that in HC who received the booster dose, but the seroconversion rate increased to 100% in the patients after the booster. Of note, the booster vaccine resulted in a significant increase of antibodies in elderly patients who had shown a response that was inferior to that in younger patients after two doses of vaccination. Therefore, vaccination more than three times may have the advantage for patients with mature T/NK-cell neoplasms, especially in elderly patients.

## Supplementary Information

Below is the link to the electronic supplementary material.

Supplementary file1 (PDF 146 kb)

Supplementary file2 (PDF 231 kb)

## Data Availability

The datasets generated and/or analyzed during the current study are available from the corresponding author upon reasonable request.
