# Supplementary material for: Humoral response to mRNA-based COVID-19 vaccine and booster effect of a third dose in patients with mature T cell and NK-cell neoplasms
Source: Ann Hematol. 2023 Mar 2;102(4):819–27. doi: 10.1007/s00277-023-05142-4 (PMC9978274; doi:10.1007/s00277-023-05142-4)
Supplement: Supplementary file 1 — Supplementary file1 (PDF 146 kb) [file 277_2023_5142_MOESM1_ESM.pdf]

Supplemental Figure 1. Anti-SARS-CoV-2 S antibody titers at 3 months and 9 months after the second vaccination in patients who were receiving or not receiving active treatment.

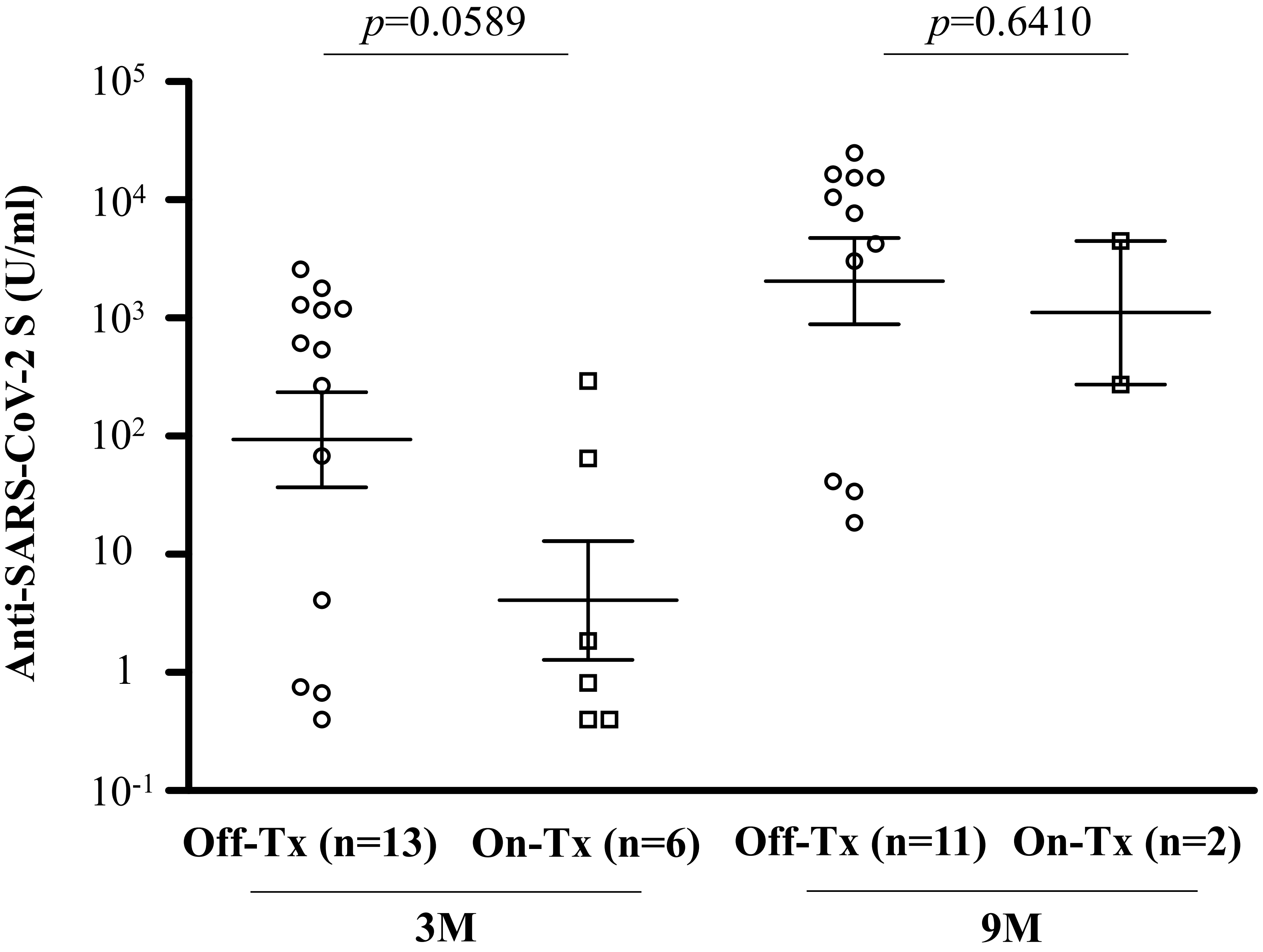

Anti-SARS-CoV-2 S antibody titers at 3 months and 9 months after the second vaccination in patients who were receiving or not receiving active treatment. Individuals who did not receive a third dose prior to the 9-month blood sampling were excluded from the analysis of 9-month blood sampling. The Mann-Whitney *U* test was used to compare medians of antibody titers. The two short lines show interquartile range (IQR) and the center long line shows the median. Off-Tx, off treatment. On-Tx, on treatment. M, months.

Supplemental Figure 2. Anti-SARS-CoV-2 S antibody titers at 3 months and 9 months after the second vaccination in patients with CR and in patients with non-CR.

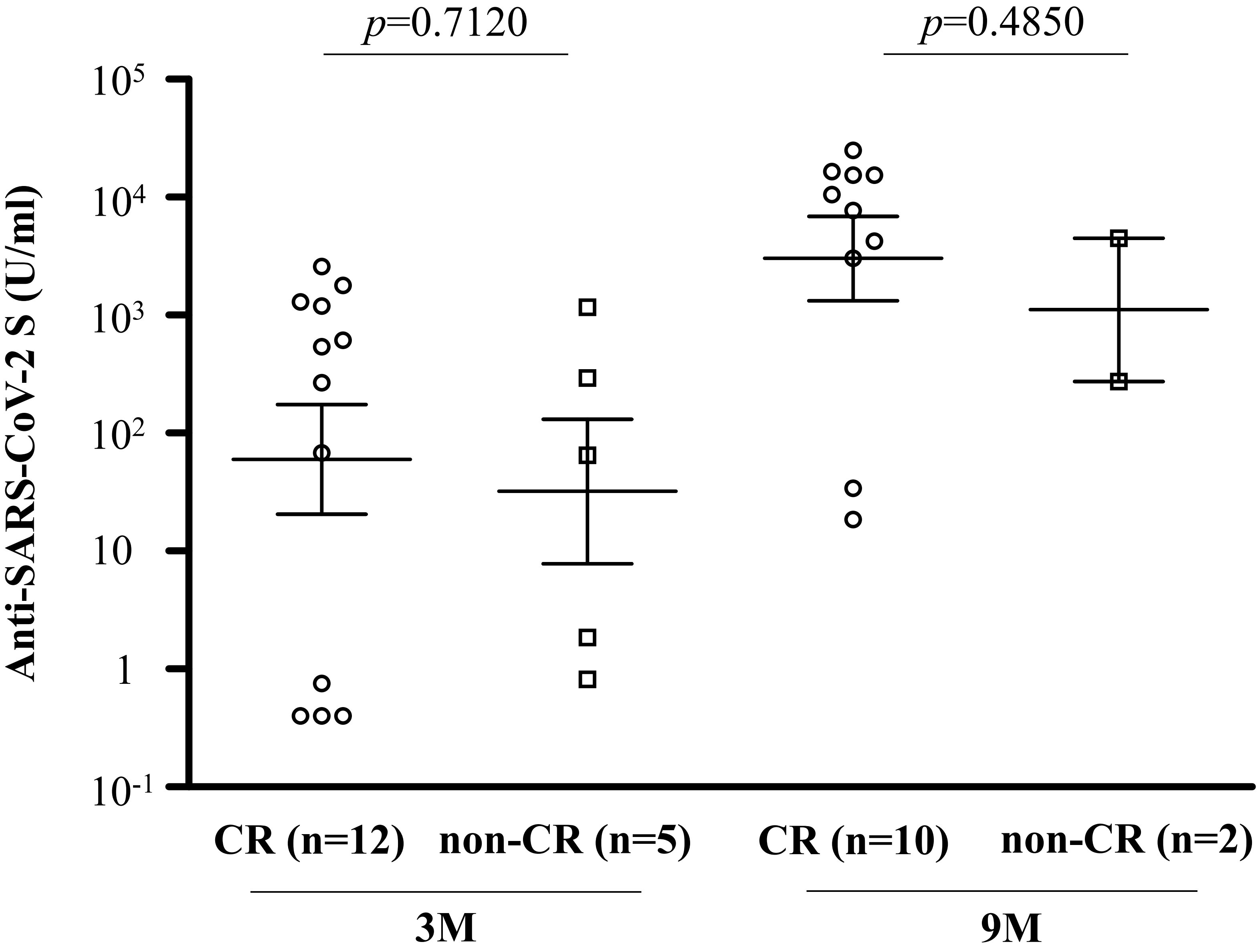

Anti-SARS-CoV-2 S antibody titers at 3 months and 9 months after the second vaccination in patients with CR and in patients with non-CR. Individuals who did not receive a third dose prior to the 9-month blood sampling were excluded from the analysis of 9-month blood sampling. The Mann-Whitney *U* test was used to compare medians of antibody titers. The two short lines show interquartile range (IQR) and the center long line shows the median.
CR, complete response. M, months.

Supplemental Figure 3. Dynamics of antibody titers in each patient.

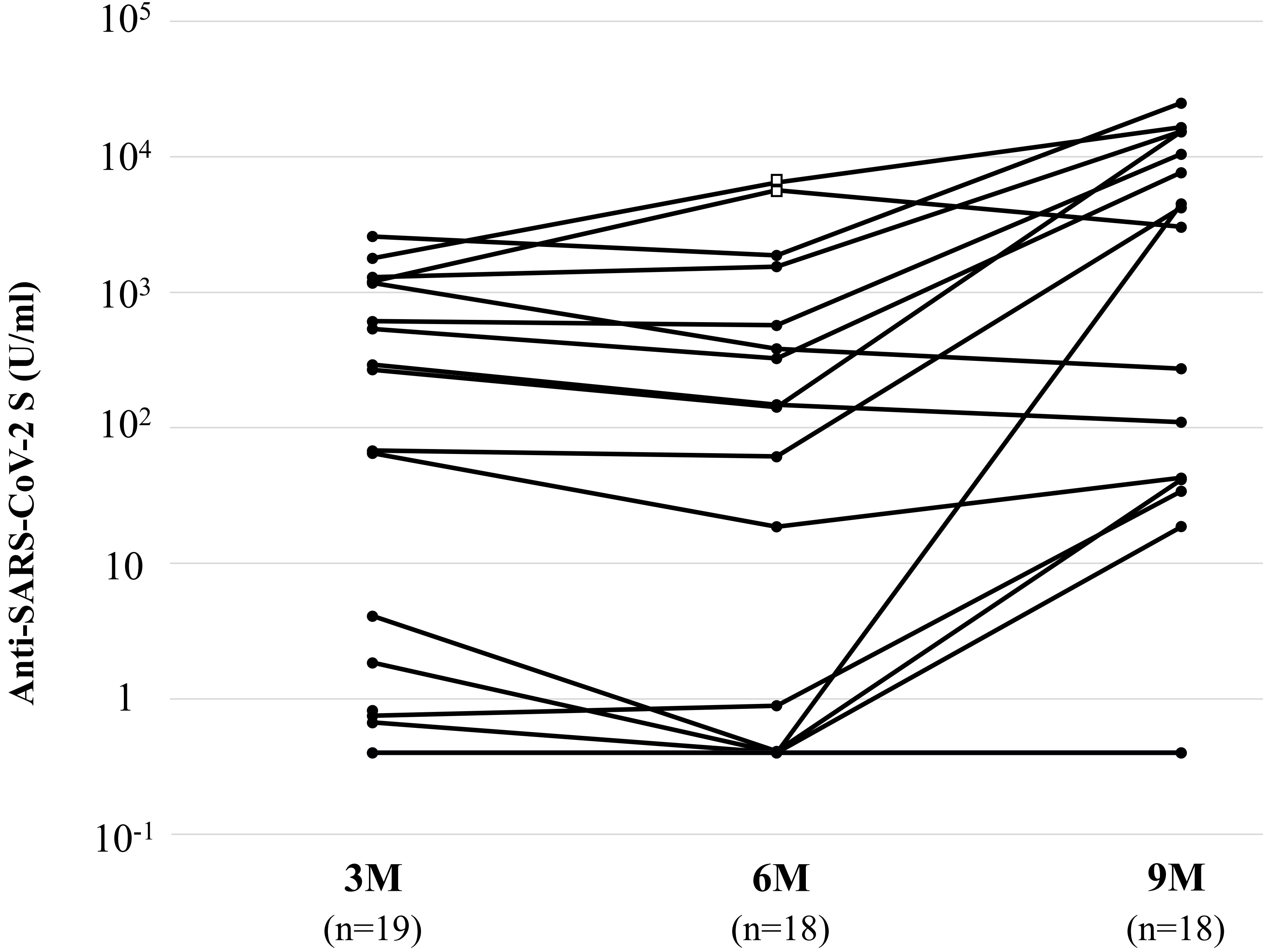

Dynamics of antibody titers in each patient. Each dot represents anti-SARS-CoV-2 S antibody titers at 3 months, 6 months, and 9 months after the second vaccination in patients. The square plots shows two patients who had received the booster vaccine before the day of 6-month blood sampling.
M, months.
