# Supplementary material for: Humoral response to mRNA-based COVID-19 vaccine and booster effect of a third dose in patients with mature T cell and NK-cell neoplasms
Source: Ann Hematol. 2023 Mar 2;102(4):819–27. doi: 10.1007/s00277-023-05142-4 (PMC9978274; doi:10.1007/s00277-023-05142-4)
Supplement: Supplementary file 2 — Supplementary file2 (PDF 231 kb) [file 277_2023_5142_MOESM2_ESM.pdf]

Supplemental Figure 1 for peer review

Anti-SARS-CoV-2 S antibody titers at 3 months after the second vaccination in healthy controls and in patients with mature T/NK-cell neoplasms under 75 years old.

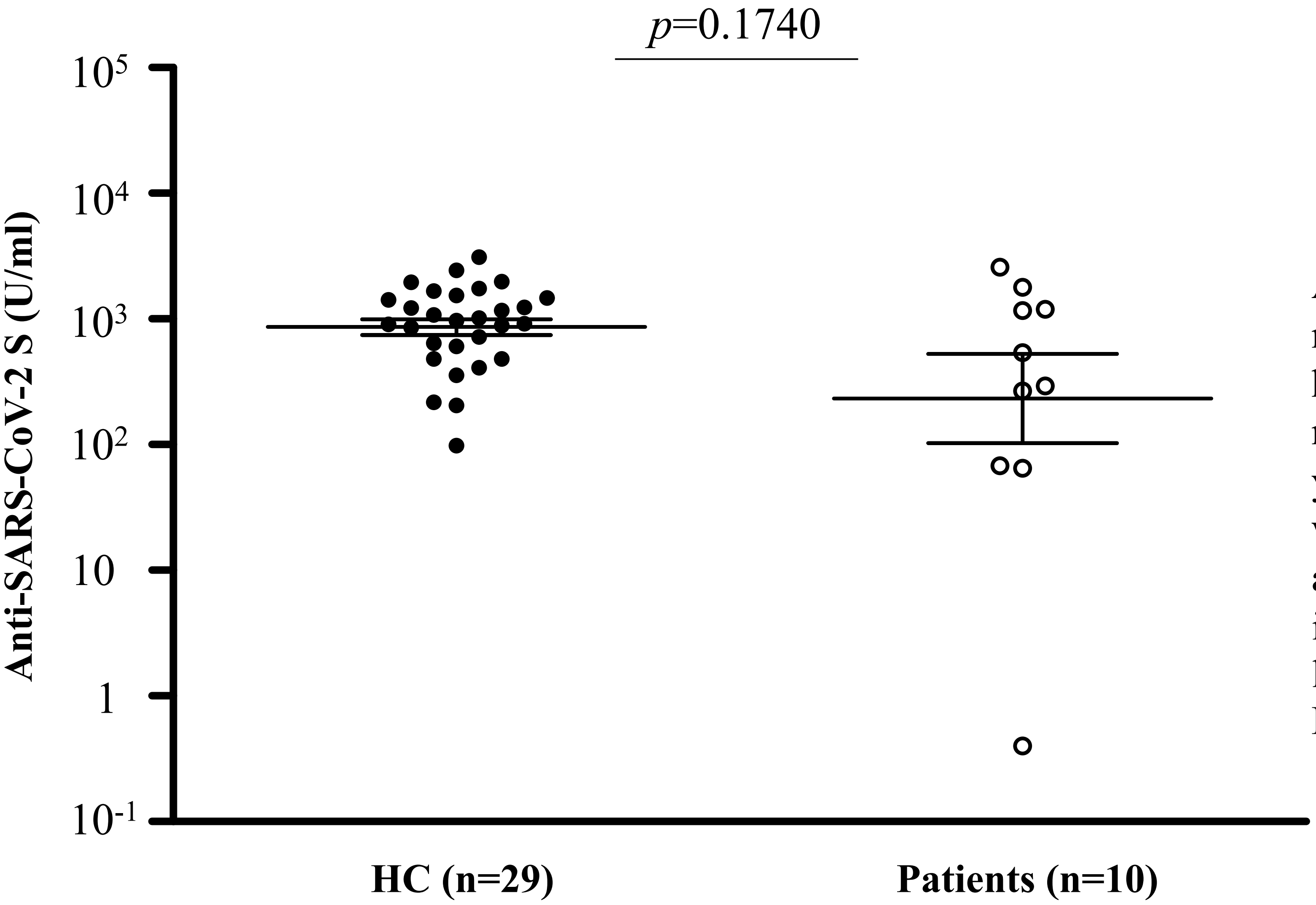

Anti-SARS-CoV-2 S antibody titers at 3 months after the second vaccination in healthy controls and in patients with mature T/NK-cell neoplasms under 75 years old. The Mann-Whitney *U* test was used to compare medians of antibody titers. The two short lines show interquartile range (IQR) and the center long line shows the median.
HC, healthy controls.

Supplemental Figure 2 for peer review

Anti-SARS-CoV-2 S antibody titers at 9 months after the second vaccination in healthy controls and patients with mature T/NK-cell neoplasms under 75 years old who received the booster vaccine and those who did not receive the booster vaccine.

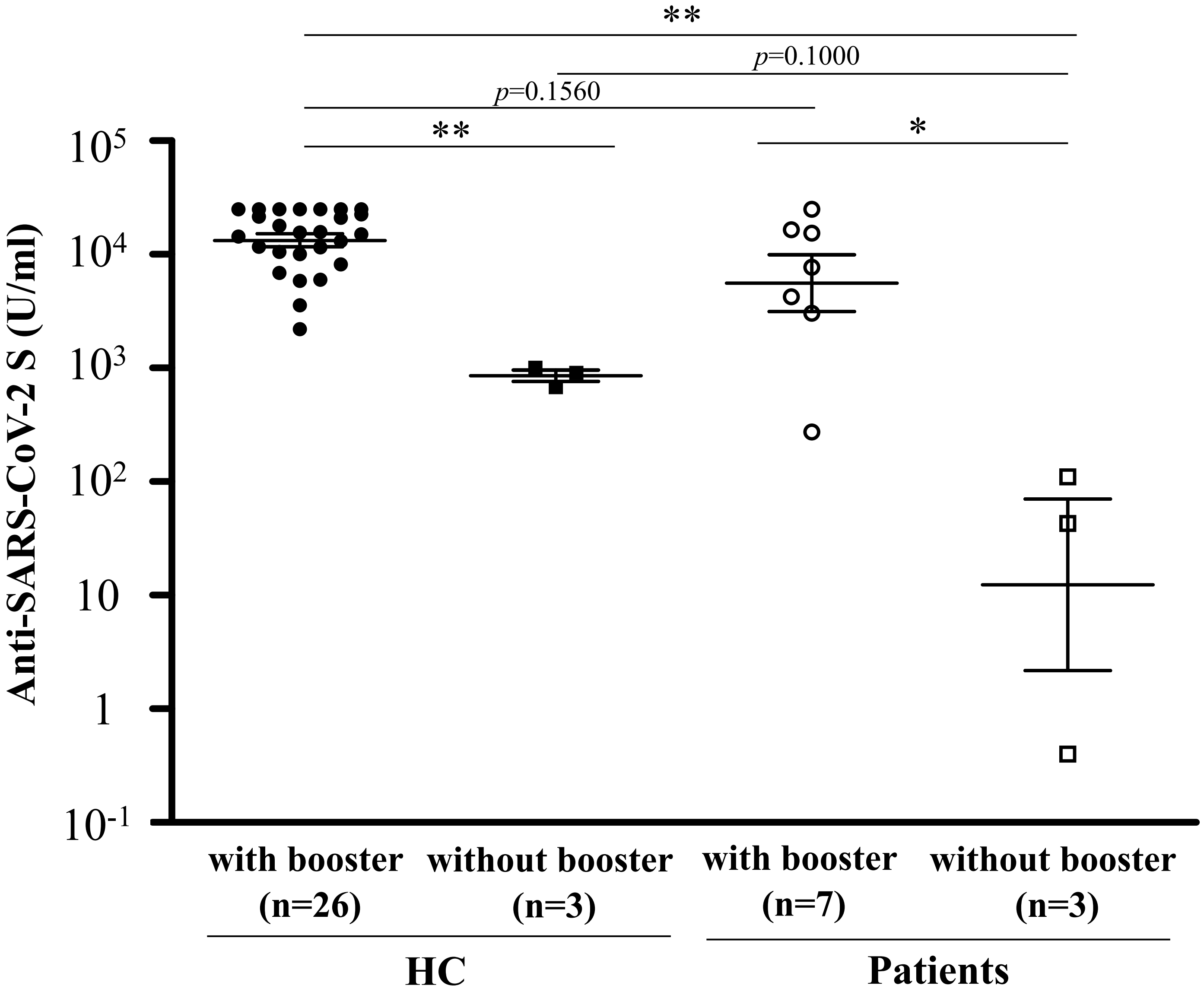

Anti-SARS-CoV-2 S antibody titers at 9 months after the second vaccination in healthy controls and patients with mature T/NK-cell neoplasms under 75 years old who received the booster vaccine and those who did not receive the booster vaccine. The Mann-Whitney *U* test was used to compare medians of antibody titers. The two short lines show interquartile range (IQR) and the center long line shows the median.
HC, healthy controls.

* $p<0.05$; ** $p<0.01$;

Supplemental Figure 3 for peer review

Anti-SARS-CoV-2 S antibody titers after the initial vaccination and booster vaccination in healthy controls and patients with mature T/NK-cell neoplasms under 75 years old.

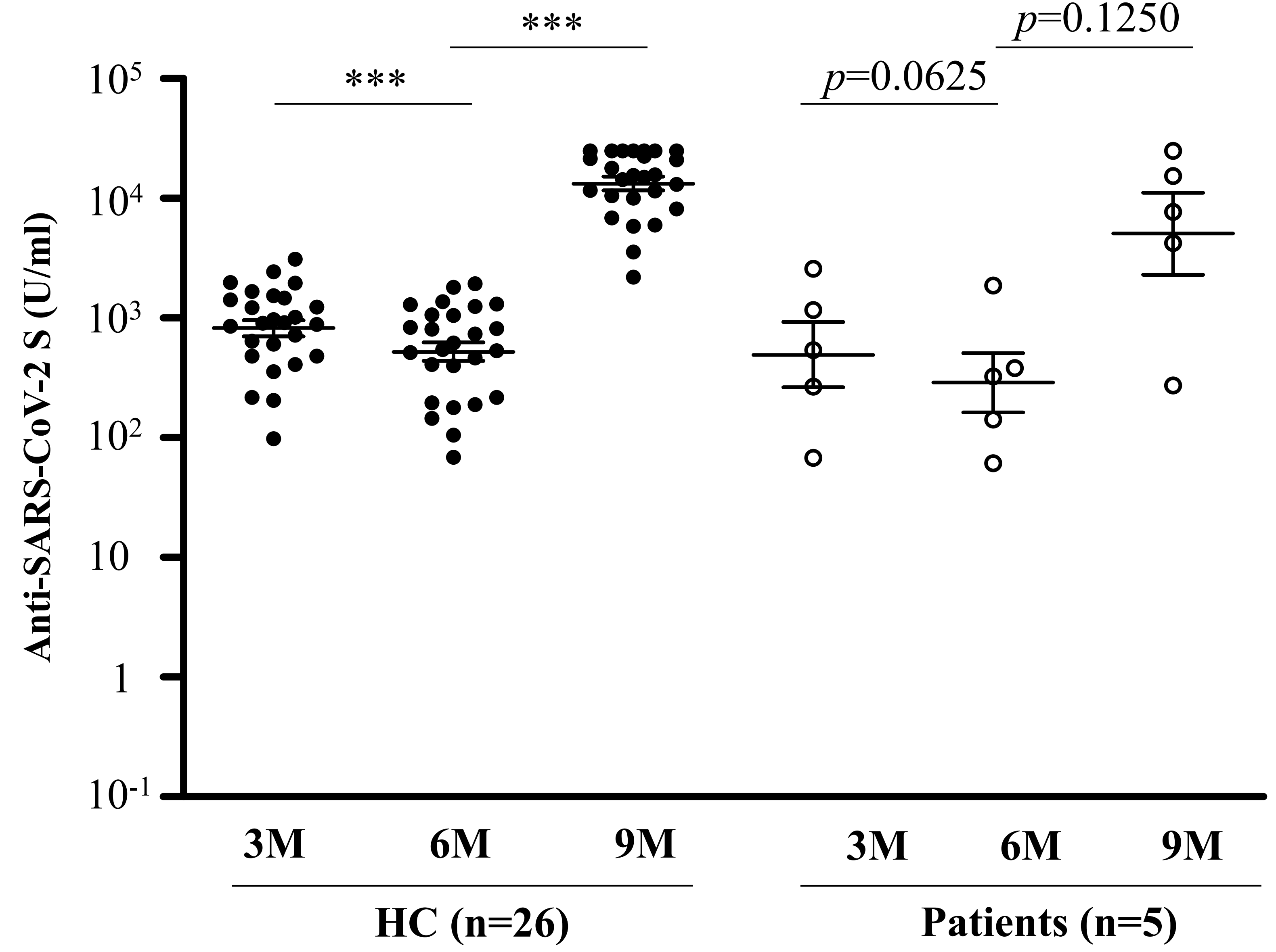

Anti-SARS-CoV-2 S antibody titers after the initial vaccination and booster vaccination in healthy controls and patients with mature T/NK-cell neoplasms under 75 years old. Individuals who did not receive a third dose were excluded from this analysis. The Wilcoxon signed-rank test was used to compare medians of antibody titers in paired individuals. The two short lines show interquartile range (IQR) and the center long line shows the median. HC, healthy controls. M, months.

** $p<0.01$; *** $p<0.001$;

Supplemental Figure 4 for peer review

Anti-SARS-CoV-2 S antibody titers at 3 months after the second vaccination in healthy controls and in patients with mature T/NK-cell neoplasms excluded patients on treatment at the time of the second vaccination.

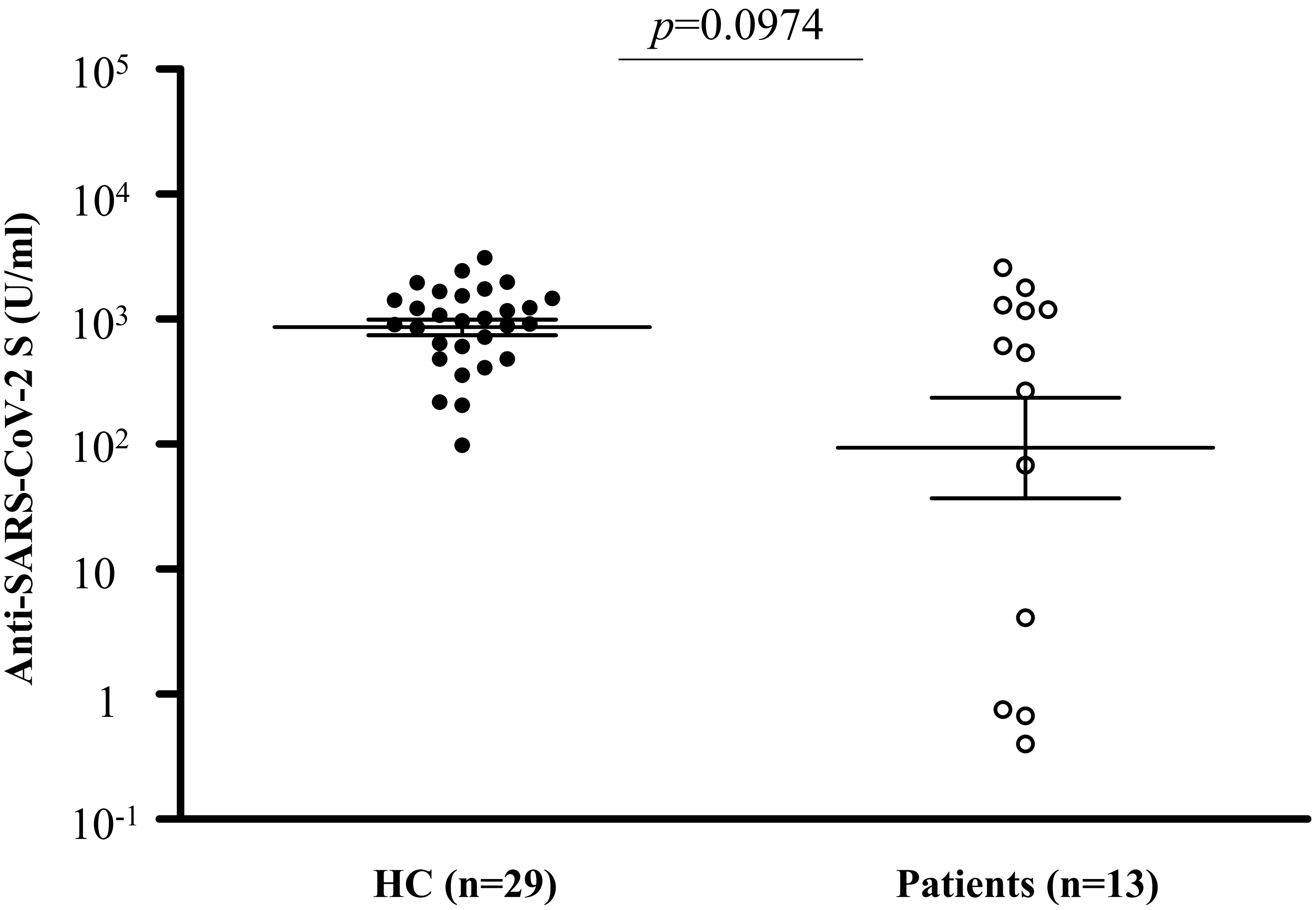

Anti-SARS-CoV-2 S antibody titers at 3 months after the second vaccination in healthy controls and in patients with mature T/NK-cell neoplasms excluded patients on treatment at the time of the second vaccination. The Mann-Whitney *U* test was used to compare medians of antibody titers. The two short lines show interquartile range (IQR) and the center long line shows the median. HC, healthy controls.

Supplemental Figure 5 for peer review

Anti-SARS-CoV-2 S antibody titers at 9 months after the second vaccination in healthy controls and patients with mature T/NK-cell neoplasms who were not receiving active treatment at the time of the second vaccination and received the booster vaccine.

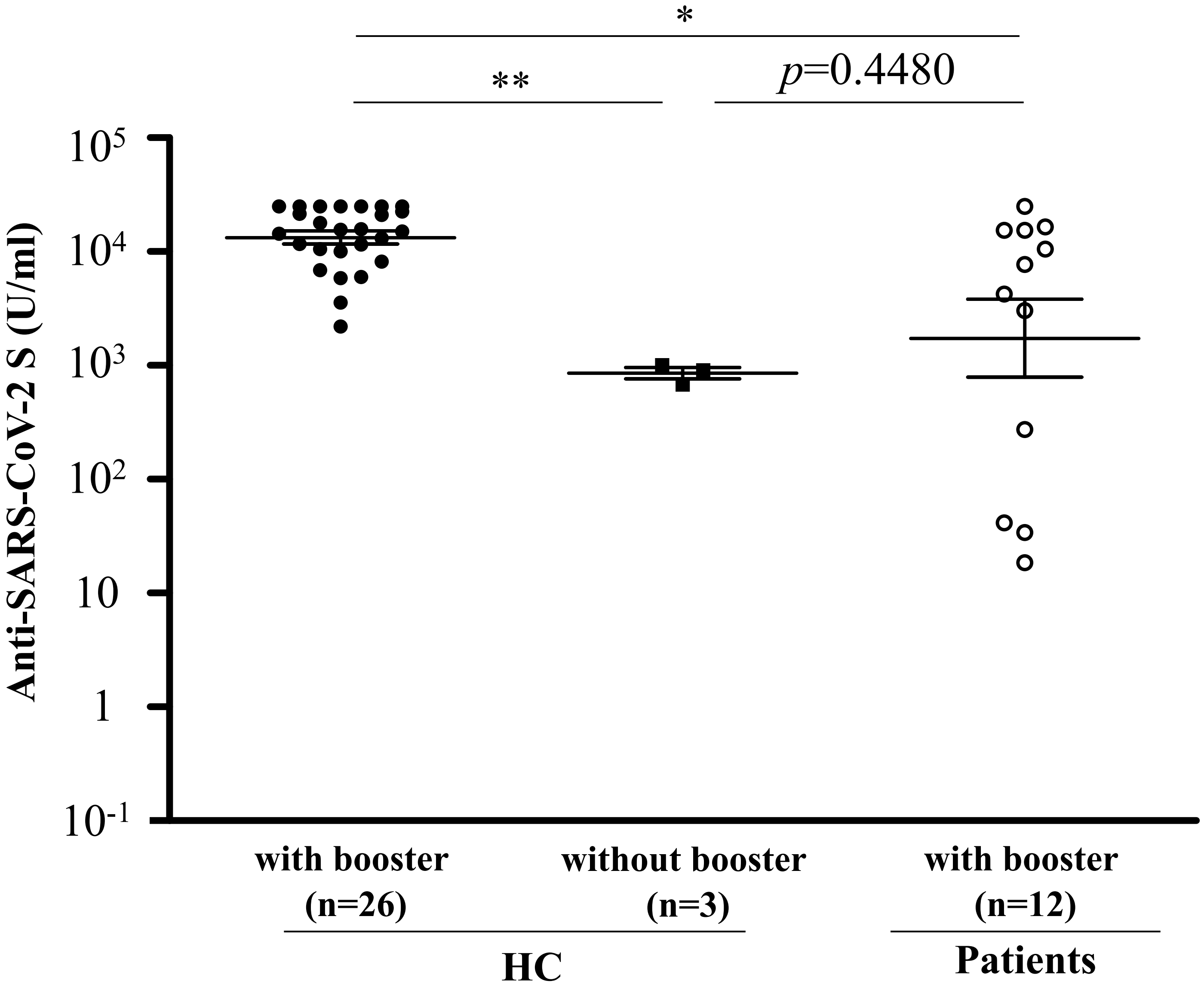

Anti-SARS-CoV-2 S antibody titers at 9 months after the second vaccination in healthy controls and patients with mature T/NK-cell neoplasms who were not receiving active treatment at the time of the second vaccination and received the booster vaccine. The Mann-Whitney *U* test was used to compare medians of antibody titers. The two short lines show interquartile range (IQR) and the center long line shows the median. HC, healthy controls.

* $p<0.05$; ** $p<0.01$;

Supplemental Figure 6 for peer review

Anti-SARS-CoV-2 S antibody titers after the initial vaccination and booster vaccination in patients with mature T/NK-cell neoplasms excluded patients on treatment at the time of the second vaccination.

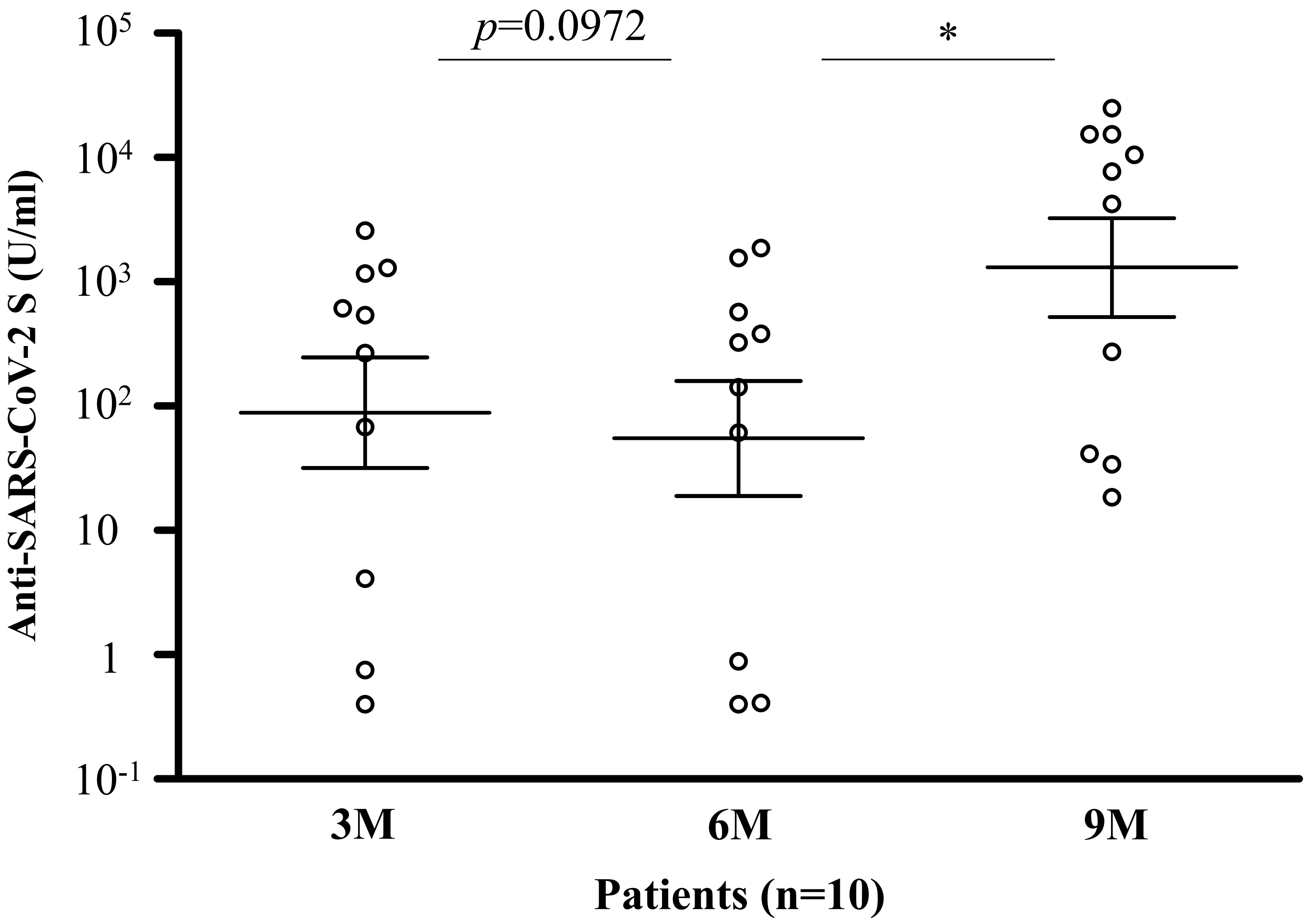

Anti-SARS-CoV-2 S antibody titers after the initial vaccination and booster vaccination in patients with mature T/NK-cell neoplasms excluded patients on treatment at the time of the second vaccination. Individuals who did not receive a third dose were excluded from this analysis. The Wilcoxon signed-rank test was used to compare medians of antibody titers in paired individuals. The two short lines show interquartile range (IQR) and the center long line shows the median. M, months.

* $p<0.05$;
